# Erratum to: Delayed Closure of Giant Omphaloceles in West Africa: Report of Five Cases

**DOI:** 10.1055/s-0038-1632382

**Published:** 2018-03-06

**Authors:** Oumama El Ezzi, Raymond Bossou, Olivier Reinberg, Sabine Vasseur Maurer, Anthony de Buys Roessingh

**Affiliations:** 1 Department of Pediatric Surgery, CURCP, Centre Hospitalier Universitaire Vaudois, Lausanne, Switzerland; 2 Departement du zou et collines – Pediatry, Abomey, Benin

It has been brought to the Publisher's attention that the author's name "Sabine Vasseur Maurer" was not tagged correctly in the above-mentioned article, published in *European J Pediatr Surg Rep* vol 5, issue 1. DOI of the original article is 10.1055/s-0037-1599796 . The names should be listed with "Sabine” as first name and “Vasseur Maurer" as family name.

